# Characterization of a novel *AEL* allele harboring a c.28 + 5G>A mutation on the ABO*A2.01 background: a study utilizing PacBio third-generation sequencing and functional assays

**DOI:** 10.3389/fimmu.2024.1396426

**Published:** 2024-12-23

**Authors:** Lin-Nan Shao, Wen-Qian Song, Lu Zhou, Ling-Zi Pan, Ying Duan, Nan Xiao, Shi-Hang Zhou, Xiao-Hua Liang

**Affiliations:** Blood Group Reference Laboratory, Dalian Blood Center, Dalian, China

**Keywords:** novel AEL allele, intron, splice site, minigene, PacBio, third-generation sequencing

## Abstract

**Background:**

Mutations in the ABO gene, including base insertions, deletions, substitutions, and splicing errors, can result in blood group subgroups associated with the quantity and quality of blood group antigens. Here, we employed third-generation PacBio sequencing to uncover a novel *AEL* allele arising from an intron splice site mutation, which altered the expected A_2_ phenotype to manifest as an Ael phenotype. The study aimed to characterize the molecular mechanism underlying this phenotypic switch

**Methods:**

A 53-year-old healthy male blood donor with an atypical agglutination pattern was investigated. PacBio sequencing was used to sequence the entire *ABO* gene of the proband. *In silico* analysis predicted aberrant splicing, which was experimentally verified using a minigene splicing assay.

**Results:**

Based on serological characteristics, the proband was determined to have an Ael phenotype. Sequencing revealed heterozygosity for ABO*O.01.02 and a novel ABO*A2.01-like allele with an additional c.28 + 5G>A mutation in intron 1. *In silico* predictions also indicated that this mutation is likely to cause aberrant splicing. Minigene analysis suggested that this mutation disrupted the 5’-end canonical donor splice site in intron 1, activated a cryptic donor site, and resulted in a 167 bp insertion, producing a truncated glycosyltransferase (p.Lys11Glufs*66). Meanwhile, a small amount of the wild type transcript was also generated through normal splicing, contributing to the Ael phenotype.

**Conclusion:**

A novel *AEL* allele was identified in a Chinese male blood donor on the ABO*A2.01 background, characterized by the c.28 + 5G>A variant. This study provides insights into the molecular basis of blood group antigen variation.

## Introduction

The ABO blood group system, discovered by Karl Landsteiner more than a century ago (1), continues to be of paramount importance in blood transfusion, prenatal serological testing, and bone marrow as well as organ transplantation. The gene responsible for the ABO system is located on chromosome 9 and codes for the glycosyltransferases A and B enzymes, which are responsible for the production of A and B antigens on red blood cells (RBCs), respectively. The *ABO* gene encompasses over 18 kb and is composed of seven exons, each ranging in size from 28 to 688 bp. Exons 6 and 7, the final two exons, make up 823 bp of the transcribed 1062 bp mRNA and are responsible for encoding the catalytic domain of the ABO glycosyltransferases (2, 3). Numerous studies have demonstrated that base insertions, deletions, substitutions, and mutations within the coding exons of the ABO gene can give rise to subgroups (4–7), which directly influence the integrity and abundance of blood group antigens (8, 9). Additionally, certain ABO subgroups exhibit weak expression and can be attributed to variants within the introns at exon/intron boundaries (10) and in regulatory regions, including the CCAAT-binding factor/Nuclear Factor Y (CBF/NF-Y) binding site (11), the proximal promoter (4), and the +5.8-kb site (12). These subgroups are frequently misclassified as common ABO genotypes due to the predominant focus on coding regions. Third-generation long-read single-molecule real-time sequencing technology, as provided by Pacific BioSciences (PacBio), holds significant potential for the comprehensive assembly of haplotype sequences of blood group gene alleles (13).

The Ael phenotype represents a rare subgroup distinguished by its absence of agglutination in reaction to anti-A or anti-A,B antibodies. Nonetheless, Ael RBCs do interact with these antibodies, as shown by adsorption and elution tests (14). Flow cytometry (15) and immunogold electron microscopy (16, 17) have revealed very low levels of A antigen on Ael cells. Individuals with the Ael phenotype typically have serum containing anti-A_1_ antibodies and may also have antibodies that agglutinate A_2_ cells (18). Notably, glycosyltransferase A is undetectable in Ael serum or on RBC membranes, and the H-transferase activity in Ael serum is less than that in A_1_ or A_2_ serum (19). It is necessary to use serological and/or molecular biology techniques to accurately identify the blood type of such patients, in order to ensure that weak antigens are not missed and avoid blood transfusion in cases of blood type inconsistency.

In this study, we employ PacBio sequencing to describe a novel *AEL* allele where an intron splice site mutation, which would normally result in an A_2_ phenotype, anomalously leads to an Ael phenotype, and we aim to elucidate its underlying mechanism.

## Materials and methods

### Specimen collection

A 53-year-old healthy male with an anomalous agglutination pattern visited our blood center to donate blood. Upon providing informed consent, a specimen was collected using EDTA anticoagulant.

### Serological tests

Serological tests were conducted, which included ABO blood typing using standard serologic methods, comprising forward and reverse typing. Forward typing employed anti-A, anti-B, anti-AB, and anti-H (anti-A and anti-B from Changchun Brother Biotech Co, Ltd., anti-AB from DIAGAST, and anti-H from Shanghai Hemo-pharmaceutical Biological Company) reagents to detect A, B, and H antigens, respectively, on red blood cells (RBCs). Reverse typing was carried out using A_1_, B, and O cells (from Shanghai Hemo-pharmaceutical Biological Company) with a tube test by qualified personnel, following the manufacturer’s guidelines. To confirm the presence of A antigens on the RBCs, adsorption and elution procedures were conducted using monoclonal anti-A antibody, following the standard protocol, with heat elution being the method employed.

### PacBio third-generation long-read single-molecule real-time sequencing

**Figure 1:**
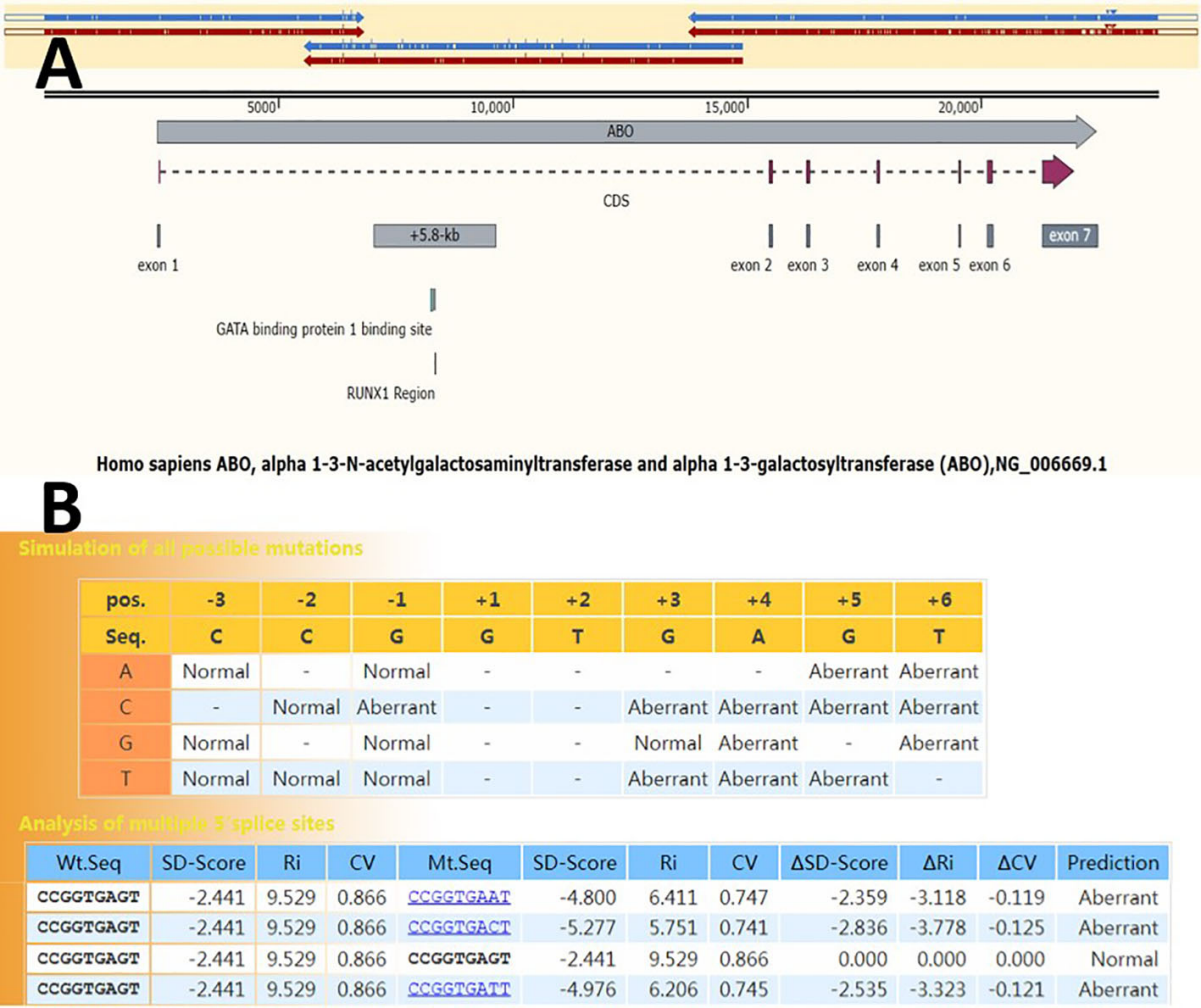
Genetic structure of the *ABO* gene. **(A)** Three overlapping segments (blue and red arrows above) of the long-range PCR amplification cover the entire ABO gene. The vertical lines inside the blue and red arrows indicate mutation sites. Full-length gene haplotypes can be assembled based on the mutation sites within the overlapping region. NG_006669.1 was used as the reference allele sequence. **(B)** SD-Score prediction results of 5’ splice site sequences spanning three nucleotides at the 3’ end of exon 1 and six nucleotides at the 5’ end of intron 1 (from exon –3 to intron +6). The program returns differences in the SD-Score (ΔSD-Score), the information contents (ΔR*i*), and the position weight matrix (ΔCV) in the simulation of all possible single nucleotide variations.

Genomic DNA was extracted from peripheral whole blood samples using a commercially available HiPure Blood DNA Mini Kit, according to the manufacturer’s instructions. For long-range PCR, reaction mixtures contained 5 μL of 5× PrimeSTAR GXL buffer, 2 μL of dNTP mixture (2.5 mM each), 0.5 μL of PrimeSTAR GXL DNA polymerase, 0.26 μL of each PCR primer mix (100µM), 30 ng DNA templates, and DNase/RNase-free deionized water, resulting in a final reaction volume of 25 μL. The PCR cycling program comprised an initial denaturation at 94°C for 2 min, followed by 26 cycles of 98°C for 12 s, 68°C for 12 min (beginning from the 11th cycle, each subsequent cycle increased by 30 s), and a final extension step at 68°C for 10 min. The PCR products were used for preparing the library for PacBio sequencing. The entire ABO gene, including flanking regulatory regions in the three overlapping fragments, was amplified. The fragments overlapped more than 1 kb (**Figure 1A**).

### In silico analysis

The effect of the splice site variation was predicted using the SD-Score web service program (https://www.med.nagoya-u.ac.jp/neurogenetics/SD_Score/sd_score.html). The frequency of a specific 5’ splice site sequence in the human genome can be used as an indicator of splicing signal intensity. The SD-Score represents a common logarithm of the frequency of a specific 5’ splice site in the human genome. For example, the SD-Score for CAG|GTGAGG, which was observed at 2562 sites in 189249 human 5’ GT splice sites, is log (2562/189249) =-1.868. The SD-Score of a splice site sequence that never appears in the human genome should be log (0/189249)= -∞ but is defined as log (0.25/189249)=-5.879 to simplify calculations. This algorithm predicts whether a mutation at the 5’ splice site will cause aberrant splicing, with a sensitivity of 97.1% and specificity of 94.7% (20). The ΔSD-Score is calculated by subtracting the wild-type SD-Score from the mutant SD-Score. Splicing patterns of potential splicing variants were predicted using the Rare Disease Data Center (RDDC) online RNA Splicer tool (https://rddc.tsinghua-gd.org/searchmiddle?to=SplitToolModel). The Berkeley Drosophila Genome Project (BDGP, http://www.fruitfly.org) splice site prediction program was employed to predict the generation and/or activation of new donor sites. 3D models of the wild type (WT) and novel mutant enzymes were generated via homology modeling with Phyre 2 (http://www.sbg.bio.ic.ac.uk/~phyre2/html/page.cgi?id=index).

### Minigene splicing assay

**Figure 2:**
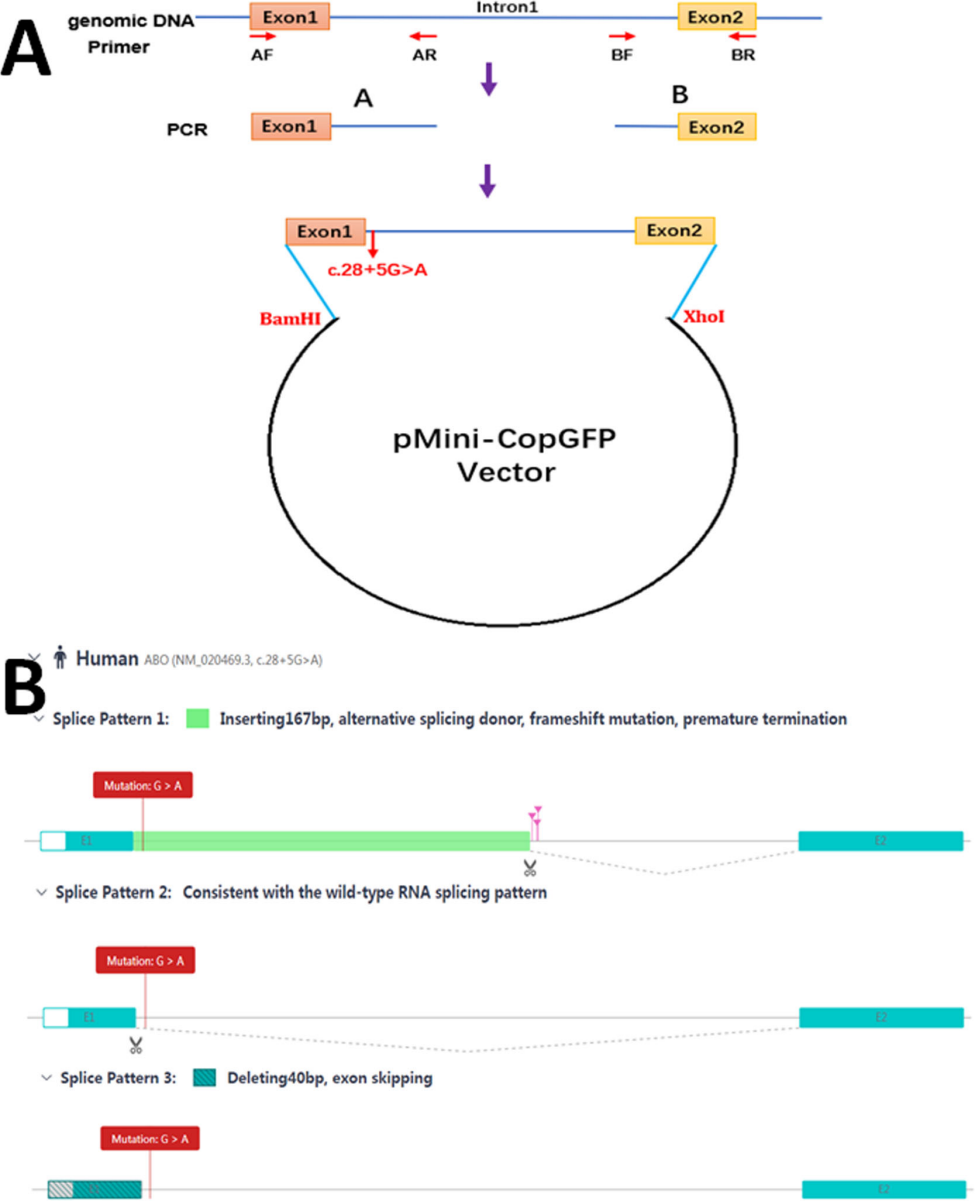
Schematic diagram for the mutation c.28 + 5G>A affecting splicing. **(A)** Plasmid construction flow chart; **(B)** The online RNA Splicer tool predicted three splice patterns: splice pattern 1 with 167 bp insertion, splice pattern 2 with the normal wild-type RNA splice, and splice pattern 3 with exon 40 bp deletion.

To investigate the potential splicing effects of the c.28 + 5G>A mutation, a minigene splicing assay was conducted *in vitro*. The minigene regions encompassing *ABO* exon 1, parts of intron 1, and exon 2 were amplified from the gDNA of the proband. Given the large size of intron 1 in the *ABO* gene, it was divided into two fragments—approximately 0.66 kb at the 5’-end and approximately 0.36 kb at the 3’-end—and these were subsequently constructed into a plasmid (**Figure 2A**).

Two sets of primers, AF/AR and BF/BR (5’-AAGCTTGGTACCGAGCTCGGATCCAGACGCGGAGCCATGGCCGAGGTGTTGC-3’/5’-CACGTCGAGGGCGCCAAGAAAATTAAGGACAGGTGATCCAC-3’ and 5’-TTCTTGGCGCCCTCGACGTGCTCATTTCAGTGTGGTTC-3’/5’-TTAAACGGGCCCTCTAGACTCGAGCCAAACAAGACCAAGACAAGCATTATTA-3’), were developed to amplify the heterozygous c.28 + 5G>A mutation site from the gDNA fragment by seamless cloning (Vazyme Biotech Co., Ltd., Nanjing, China). The PCR products were then recombined and cloned into the two digestion sites (BamHI/XhoI) of the pMini-CopGFP vector (Hitrobio Biotechnology Co., Ltd., Beijing, China). The integrity of the WT and mutant plasmids was validated by Sanger sequencing. Subsequently, the plasmids were transfected into HEK293 cells. Total RNA was extracted from cells cultured for 48 h using a TRIzol reagent (Invitrogen, USA). RT-PCR was performed using the specific primer pair (5’-GGCTAACTAGAGAACCCACTGCTTA-3’ and 5’-CCAAACAAGACCAAGACAAGCATTA-3’). Afterward, the cDNA products were analyzed by 1% agarose gel electrophoresis and further validated through Sanger sequencing.

## Results

### Serological results

The RBCs of the proband showed no response to anti-A, anti-B, anti-A,B, or anti-A_1_, but exhibited strong agglutination (4+) when exposed to anti-H. The plasma of the proband demonstrated 1+ intensity agglutination with A_1_ cells, and 4+ intensity agglutination with B cells. The presence of A antigens on the proband’s RBCs was confirmed by the absorption-elution test (**Table 1**). The Ael phenotype is characterized RBCs that do not agglutinate with anti-A or anti-A,B antibodies, yet A antigens can be detected through absorption-elution testing. Additionally, the presence of anti-A_1_ antibodies in the serum further supports the classification of the proband as having an Ael phenotype.

**Table 1:** Results of serologic grouping.

Forward typing					Reverse typing			Adsorption and elution
Anti-A	Anti-B	Anti-A_1_	Anti-A,B	Anti-H	A_1_ cell	B cell	O cell	A_1_ cell
0	0	0	0	4+	1+	4+	0	1+

### PacBio sequencing results

**Figure 3:**
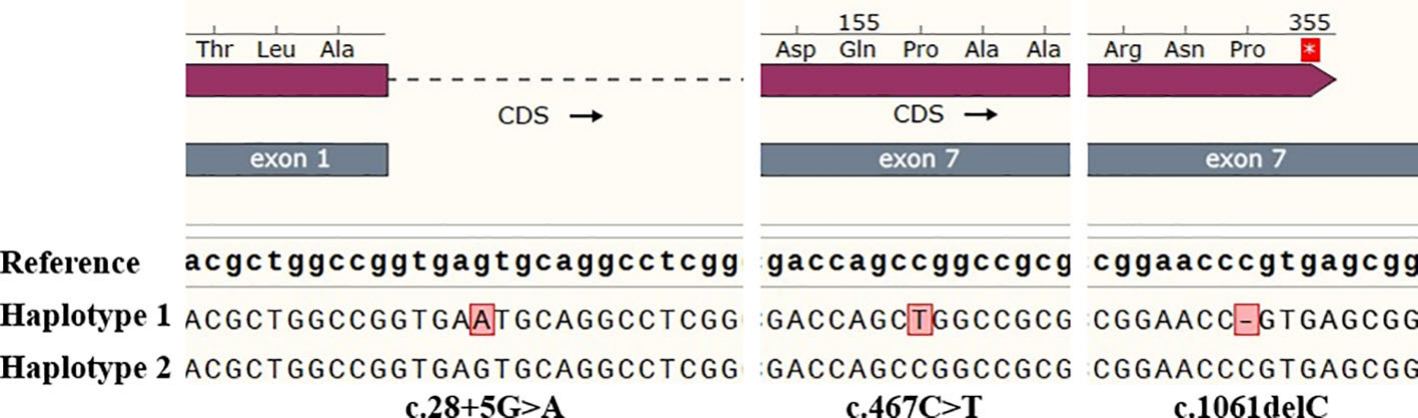
The nucleotide sequences around the mutations c.28 + 5G>A, c.467C>T and c.1061delC of the proband. Analyze through SnapGene v6.0.2 software.

Through third-generation PacBio sequencing technology, two haplotypes of the full-length ABO gene were obtained. **Table 2** summarizes the sequencing results performed in this study. The proband was found to be heterozygous for the ABO*O.01.02 and a novel ABO*A2.01-like allele. This new allele harbored an additional mutation, c.28 + 5G>A, in intron 1 (**Figure 3**). No variants were detected in the CBF/NF-Y binding site, proximal promoter, and +5.8-kb site when compared to the normal ABO*A2.01 allele.

**Table 2:** PacBio single-molecule real-time sequencing results of *ABO* gene conducted in this study.

Sample	Gene	Haplotype 1			Haplotype 2			Phenotype
		Phenotype	Allele	Mutation	Phenotype	Allele	Mutation	
Proband	*ABO*	O	ABO*O.01.02	c.106G>T; c.188G>A; c.189C>T; c.220C>T; c.261delG; c.297A>G; c.646T>A; c.681G>A; c.771C>T; c.829G>A	Ael	ABO*AEL.NEW	c.28 + 5G>A; c.467C>T; c.1061delC	Ael

### Bioinformatics analysis

A 5’ splice site with a △SD-Score greater than −0.34 is considered not to affect pre-mRNA splicing. Conversely, a mutant site with a △SD-Score less than −0.34 and an SD-Score less than −2.9 indicates abnormal splicing. If the △SD-Score is less than −0.34 but the D-Score is above −2.9, the △R*i* value is used. A △R*i* value greater than −1.45 suggests normal splicing, whereas a value less than −1.45 indicates abnormal splicing. The splicing prediction consequences of the c.28 + 5G>A variant, as determined using the SD-Score algorithm, are shown in **Figure 1B**. The △SD-Score and △R*i* value for this variant were −2.359 and −3.118, respectively, indicating that the c.28 + 5G>A variant, located at the exon/intron boundary, is likely to cause aberrant splicing. Three potential splice patterns were predicted using the online RNA splicer tool: splice pattern 1 with 167 bp insertion, splice pattern 2 with the normal WT RNA splice, and splice pattern 3 with 40 bp deletion and exon skipping (**Figure 2B**). Bioinformatics analysis with BDGP showed that the c.28 + 5G>A variant decreased the score of the canonical 5’ donor splice site from 0.99 to 0.00. The predicted new donor sites downstream are presented in **Supplementary Figure S1**.

### Minigene splicing assay

**Figure 4:**
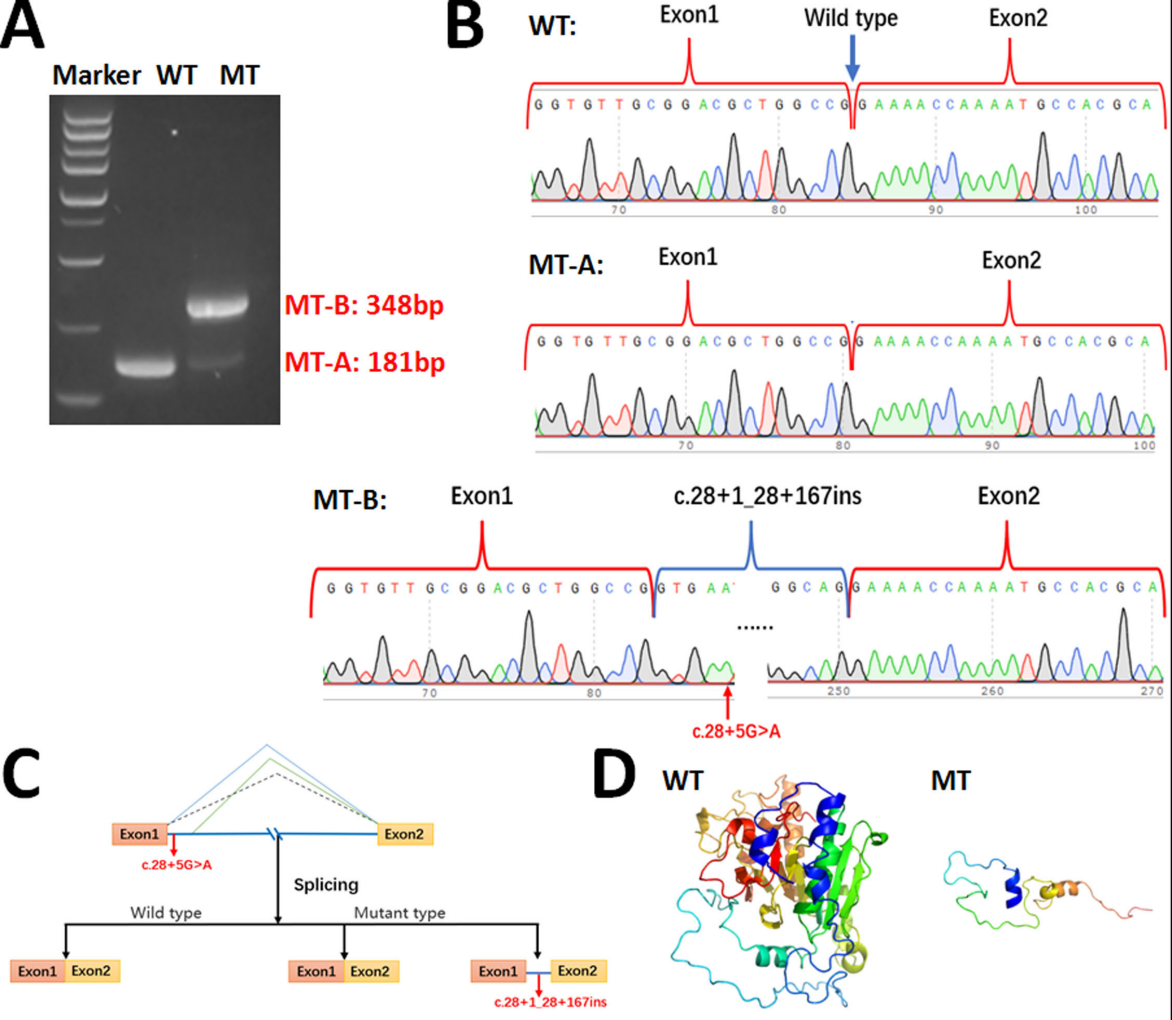
Minigene assay for *ABO* c.28 + 5G>A variant and schematic diagram of splicing pattern. **(A)** Gel electrophoresis of RT-PCR revealed a single band for wild type (181bp) and two bands for mutant type (181bp & 348bp). **(B)** Minigene product sequencing. The wild type minigene formed normal mRNA, but the c.28 + 5G>A variant of *ABO* caused normal splicing (MT-A) and abnormal splicing (MT-B), which abrogate the intron 1 canonical splice site and lead to activating a cryptic donor splice site downstream of intron 1 in the *ABO* gene and lead to a 167 bp insertion; **(C)** Schematic of splicing for *ABO* c.28 + 5G>A. **(D)** The wild type and truncated glycosyltransferase A.

We performed minigene analysis on both the WT and mutant type (MT) carrying the *ABO* c.28 + 5G>A mutation to further elucidate the nature of the abnormal splicing. Agarose gel electrophoresis of RT-PCR products demonstrated a single band for the WT, corresponding to a product of 181 bp, and two bands for the MT (MT-A: 181 bp and MT-B: 348 bp), with the MT-A band being significantly less intense than the MT-B band (**Figure 4A**). Sanger sequencing confirmed normal splicing for the WT and MT-A, whereas the MT-B exhibited abnormal splicing (**Figure 4B**), consistent with splice patterns 1 and 2 predicted by the online RNA Splicer tool and proximate donor site downstream predicted by the BDGP with a score of 0.40. The minigene analysis suggested that the c.28 + 5G>A variant can abolish the intron 1 5’-end canonical donor splice site and activate a cryptic donor splice site downstream of intron 1 in the *ABO* gene. This results in a 167 bp insertion in the mRNA leading to a truncated glycosyltransferase (p.Lys11Glufs*66) (**Figure 4C**). Amino acid sequence alignment results are provided in **Supplementary Figure S2**. Additionally, the crystallization of the WT and mutant glycosyltransferase A are presented (**Figure 4D**).

## Discussion

As of now, only eight *AEL* alleles, namely ABO*AEL.01–08, have been documented on the ISBT website (https://www.isbtweb.org/resource/001aboalleles.html, accessed Feb 26, 2024). All these alleles possess mutations within exon 7, except for ABO*AEL.04, similar to the novel allele described in this study, which also exhibits mutation at the intronic splicing site.

Pre-mRNA splicing is a fundamental process in eukaryotic gene expression, involving the accurate removal of introns and the linking of exons to produce mature mRNA. The successful completion and regulation of this process hinge on intricate biochemical interactions between *cis*- and *trans*-acting elements, including the 5’ and 3’ splice sites. Mutations at these sites can disrupt the authentic exon/intron boundary, modifying the spliceosome’s binding site and leading to abnormal splicing (21). The novel *AEL* allele identified in the proband, characterized by a mutation at the 5’ splice site of intron 1 (c.28 + 5G>A), was found to be associated with the Ael phenotype. *In silico* prediction analyses suggested that replacing the G at position c.28 + 5 with A, C, or T would result in altered splicing (**Figure 1B**).

To elucidate the mechanism underlying the c.28 + 5G>A mutation’s contribution to the Ael phenotype, we performed minigene assays. These experiments demonstrated that the mutation primarily abolishes the canonical donor site, causing a shift in the splice site towards the 3’ end and resulting in premature translation termination at position Leu75. This leads to the production of a truncated glycosyltransferase A lacking the catalytic domain, thereby affecting enzyme function relative to the WT glycosyltransferase A. Concurrently, the MT plasmid appeared to produce a minor amount of the WT transcript through normal splicing. Premature termination codons bearing transcript can be degraded by nonsense-mediated mRNA decay which is generally thought to be a eukaryotic mRNA surveillance pathway (22). However, WT transcript can generate trace amounts of functional glycosyltransferase A. This mechanism may account for the development of the Ael phenotype associated with c.28 + 5G>A. The results of the minigene assays are consistent with the aberrant splicing predicted by the SD-Score algorithm, the splice patterns 1 and 2 predicted by the online RNA Splicer tool, and the proximate donor site (score: 0.40) downstream predicted by the BDGP. Splicing site mutations can lead to exon skipping (23), however, minigene assays did not find evidence that the c.28 + 5G>A mutation causes the skipping of exon 1 (splice pattern 3) in this study.

Although the c.28 + 5G>A mutation has been previously reported (24), it differs from our findings, as it is cis-formed with ABO*B.01 rather than ABO*A2.01, leading to the Bel phenotype. However, their article did not conduct functional experiments to elucidate the mechanism. The ABO*AEL.04 allele has been reported to harbor a mutation at the 5’ splice site in intron 6 (c.374 + 5G>A) which consequently leads to the Ael phenotype (23, 25). This mutation is distinct from our finding of intron retention and instead causes exons skipping. Notably, our discovery of a mutation within the ABO*A2.01 backbone alters the expected A_2_ phenotype to an Ael phenotype, or more accurately, an A_2_el phenotype. Given the substantially reduced density of A antigen sites on A_2_ red blood cells compared to A_1_ RBCs (26), additional experimental validation is required to ascertain whether the A antigen sites on the A_2_el RBCs are indeed fewer than those on the Ael RBCs.

Our study offers several advantages. Firstly, we employed third-generation sequencing technology, which has not yet been widely popularized in the field of blood grouping, to capture two haplotypes containing the full-length and flanking regulatory regions of the *ABO* gene without ignoring mutations outside the coding region. Secondly, we identified a novel *AEL* allele and provided the first insight into the mechanism by which the c.28 + 5G>A mutation disrupts normal splicing, utilizing minigene technology. The nucleotide sequence of this new *AEL* allele has been submitted to the GenBank database (accession number: OR995727).

## Conclusions

In conclusion, we identified a novel *AEL* allele in a Chinese male blood donor. This novel allele harbors the c.28 + 5G>A variant within the ABO*A2.01 allele background, which primarily causes abnormal splicing and 167 bp insertion in the mRNA, leading to the production of a truncated glycosyltransferase (p.Lys11Glufs*66).

## Data Availability

The data presented in the study are deposited in the GenBank database, accession number OR995727. The direct link is https://www.ncbi.nlm.nih.gov/nuccore/OR995727.

## Supplementary material

Supplementary Figure 1 Prediction results of donor sites using BDGP. (A) Splice site predictions for wild type with donor score cutoff 0.40 (exon/intron boundary shown in larger font). This server runs the NNSPLICE 0.9 version (January 1997) of the splice site predictor. The correlation coefficient (CC) for donor site prediction in an optimized network with one layer of hidden units is 0.855 versus 0.810 for a network with no hidden units; for the acceptor site prediction, the values are 0.824 and 0.750 respectively. At the 5% false positive level, 6% of the real donor sites and 9% of the real acceptor sites are missed. (B) The wild type sequence used to predict the results of donor sites. The nucleotides of exon 1 are shown in uppercase. The initiation codon ATG is underlined and the c.28 + 5G is marked in red. WT, wild type; MT, mutant type.

Supplementary Figure 2 The amino acid sequence alignment results. The red part represents Helix, and the blue part represents Strand. WT, wild type; MT, mutant type. Images are generated by online web tool Novopro (https://www.novopro.cn/tools/secondary-structure-prediction.html).
