# Supplementary figures and images for: Characterization of a novel AEL allele harboring a c.28 + 5G>A mutation on the ABO*A2.01 background: a study utilizing PacBio third-generation sequencing and functional assays
Source: Front Immunol. 2024 Dec 23;15:1396426. doi: 10.3389/fimmu.2024.1396426 (PMC11701149; doi:10.3389/fimmu.2024.1396426)

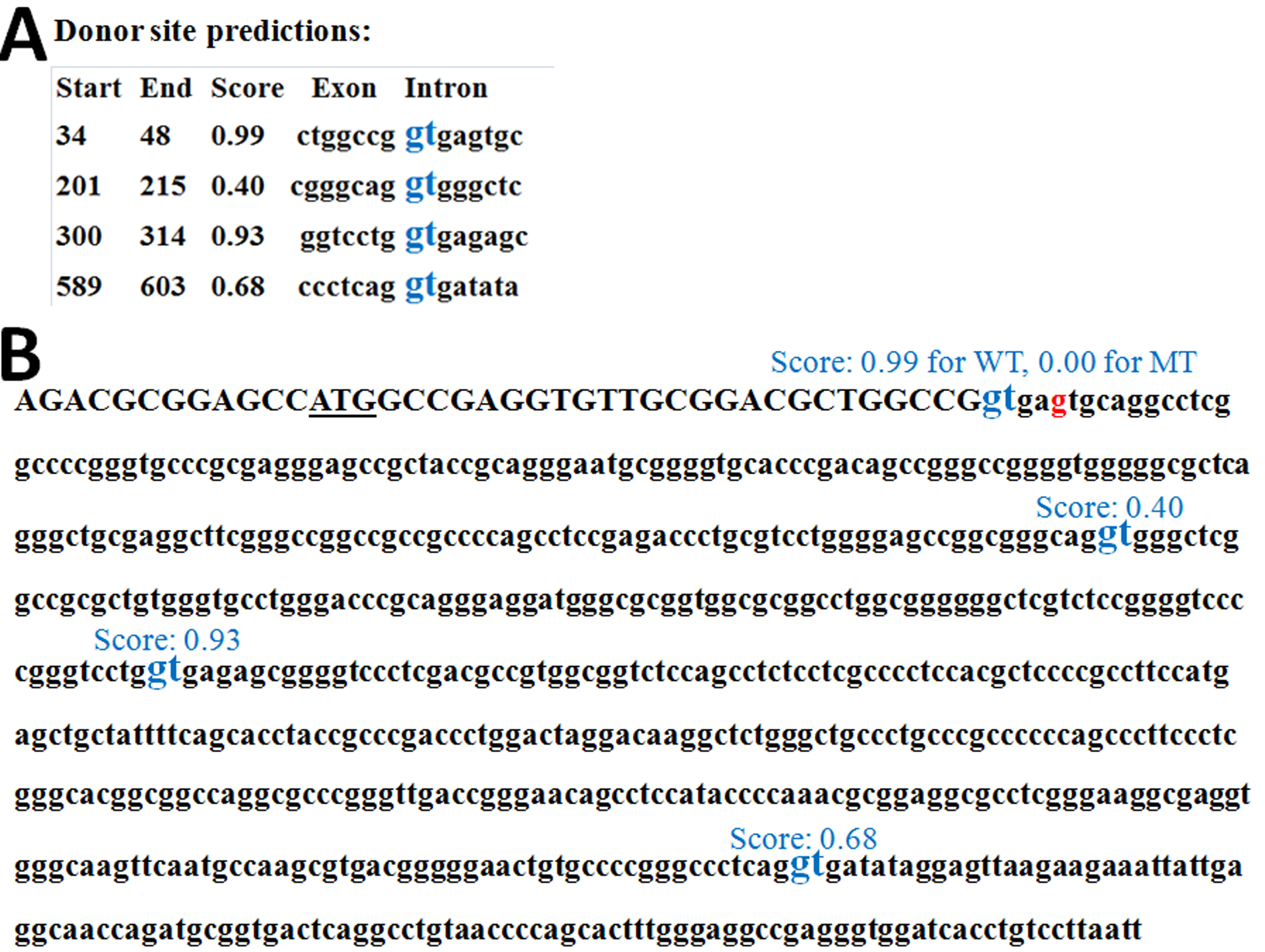

Supplement: Supplementary Figure 1 — Prediction results of donor sites using BDGP. (A) Splice site predictions for wild type with donor score cutoff 0.40 (exon/intron boundary shown in larger font). This server runs the NNSPLICE 0.9 version (January 1997) of the splice site predictor. The correlation coefficient (CC) for donor site prediction in an optimized network with one layer of hidden units is 0.855 versus 0.810 for a network with no hidden units; for the acceptor site prediction, the values are 0.824 and 0.750 respectively. At the 5% false positive level, 6% of the real donor sites and 9% of the real acceptor sites are missed. (B) The wild type sequence used to predict the results of donor sites. The nucleotides of exon 1 are shown in uppercase. The initiation codon ATG is underlined and the c.28 + 5G is marked in red. WT, wild type; MT, mutant type. [file Image1.tif]

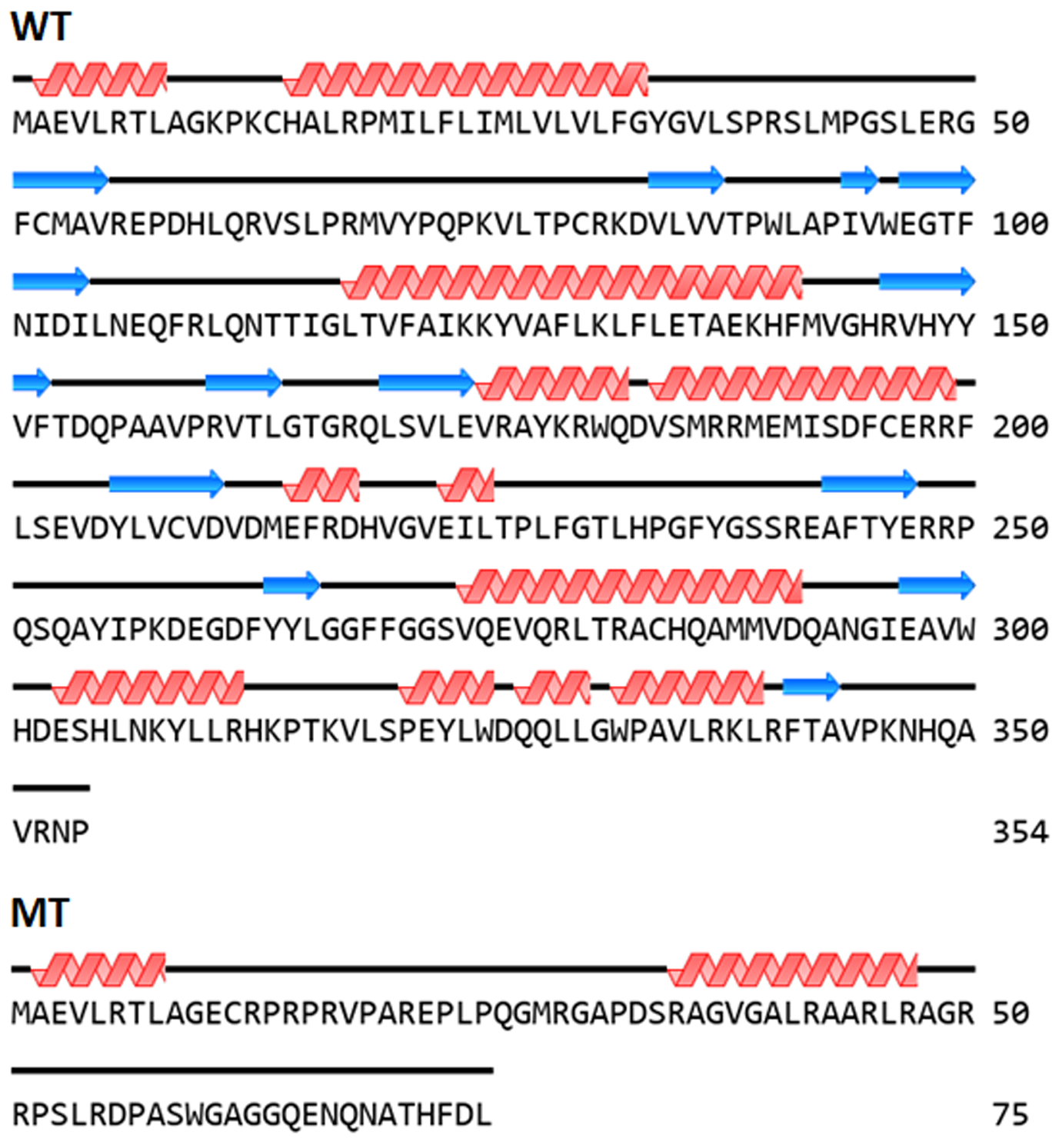

Supplement: Supplementary Figure 2 — The amino acid sequence alignment results. The red part represents Helix, and the blue part represents Strand. WT, wild type; MT, mutant type. Images are generated by online web tool Novopro (https://www.novopro.cn/tools/secondary-structure-prediction.html). [file Image2.tif]
